# Impact of health information prescription in thyroid cancer

**DOI:** 10.5195/jmla.2026.2191

**Published:** 2026-04-01

**Authors:** Marta Bianchini, Giulia Puliani, Virginia Scarinci, Gaetana Cognetti, Rosa Lauretta, Marilda Mormando, Francesca Servoli, Marialuisa Appetecchia

**Affiliations:** 1 marta.bianchini@ifo.it, Oncological Endocrinology Unit, IRCCS Regina Elena National Cancer Institute, Rome, Italy; 2 giulia.puliani@ifo.it, Oncological Endocrinology Unit, IRCCS Regina Elena National Cancer Institute, Rome, Italy; 3 virginia.scarinci@ifo.it, Digital Library R. Maceratini, IRCCS Regina Elena National Cancer Institute, Rome, Italy; 4 gaetana.cognetti@ifo.it, Digital Library R. Maceratini, IRCCS Regina Elena National Cancer Institute, Rome, Italy; 5 rosa.lauretta@ifo.it, Oncological Endocrinology Unit, IRCCS Regina Elena National Cancer Institute, Rome, Italy; 6 marilda.mormando@ifo.it, Oncological Endocrinology Unit, IRCCS Regina Elena National Cancer Institute, Rome, Italy; 7 francesca.servoli@ifo.it, Digital Library R. Maceratini, IRCCS Regina Elena National Cancer Institute, Rome, Italy; 8 marialuisa.appetecchia@ifo.it, IRCCS Regina Elena National Cancer Institute, Rome, Italy

**Keywords:** Information prescription, thyroid cancer, hospital library, psychological health, mental well-being, disease self-management, health librarianship

## Abstract

**Objectives:**

Information prescriptions consist in making specific health care information available to patients on their disease from accredited sources, in order to help them understand and manage their disease, stimulating informed participation in health care. In the literature, a few studies have investigated the real effectiveness of prescribing information on the health management of oncological diseases. The scope of our pilot study was to investigate the effectiveness of information prescription, evaluating both patient satisfaction and perception, but also its possible impact on adherence to follow-up programs.

**Methods:**

Prospective pilot study enrolling patients with thyroid cancer. They received informative scientific material on thyroid cancer, dispensed by clinical librarians of the Institute's Library.

**Results:**

101 patients were enrolled (81% were women with a total mean age of 49.39 years). 26% of patients accessed the institute's library receiving patient information materials. Comparing data of people who completed the project with those who did not, no differences in sex, age and tumour characteristics were found. We found no statistical differences in terms of adherence to follow-up visits between the two groups of patients, but health information was able to effectively respond to the requests or needs of most patients. Participating patients have improved awareness and knowledge of their disease, patient-doctor relationships, adherence to treatment and communication with family members regarding their disease status, with a final positive impact on one's psychological well-being and global satisfaction obtained in 65% of people.

**Conclusions:**

This is the first Italian study carried out in the field of oncological endocrinology, demonstrating the positive role health information has on patients' psychological health and thyroid cancer knowledge.

## INTRODUCTION

Health information prescription refers to providing specific evidence-based health information to patients with a diagnosed condition to help them understand and manage their own disease, thus stimulating informed participation in healthcare. [1–3] A health information prescription is written by the doctor, given to patients and/or family members, encouraging them to consult qualified and reliable scientific information on their diseases [4], based on the individual needs and specific requests of the patient [5].

This practice aims to inform the patient about their disease, allowing them to gain better knowledge of treatments and follow-up programs, with the potential to improve adherence and effectiveness. Furthermore, appropriate health information could change health behaviors and help patients make better, more informed healthcare decisions [6]. Knowledge is a critical factor in decision-making, it can improve patient health status and quality of life [7–10] and enhance doctor-patient communication [11, 12].

The practice of health information prescription has a long history [13, 14] but only recently, many governmental agencies across different countries have promoted models and materials for information prescription programs. [13] To date, most studies on health information prescription published in literature have mainly focused on chronic diseases, such as diabetes mellitus, hypertension or other metabolic diseases that can benefit from lifestyle modifications [15–18].

Most studies on information prescription were small pilot projects or case reports aimed at investigating patient or physician satisfaction through the use of surveys [12, 13, 19–28], while other clinical outcomes have not been well studied. In particular, analysis of the effectiveness of information prescription on health improvement or changes in self-management of diseases are scarce [4]. A qualitative semi-experimental study on 61 women with breast cancer, showed that after providing health information prescriptions, women improved their self-care capacity according to a questionnaire given to patients [29]. In another study of 21 diabetic patients, it was shown that information prescription improved medical services utilization, patients’ knowledge on their disease, and increased the capacity for self-care, enhanced disease management and strengthened the doctor-patient relationship [30].

Finally, some studies demonstrated that written information prescription is effective for minimizing healthcare costs [31–35]. A recent scoping review [4] published in 2022, reported that to date, there have been no studies conducted in Italy regarding health information prescriptions and that information prescription documents primarily address non-oncological health conditions. Furthermore, most of the studies published do not specifically report the exact steps involved in information prescription services, nor the specific roles of the members of the information prescription team, such as doctors, librarians and nurses.

To overcome these limitations, we conducted an Italian pilot study to evaluate the effect of providing health information prescriptions to thyroid cancer patients, since it is the most frequent endocrine neoplasia and it negatively impacts quality of life and psychological well-being [36–38]. This study aims to:

- identify any factors (demographic or disease-related) that could influence patient participation in a health information prescription program;
- evaluate whether health information prescription could increase patient adherence to follow-up programs;
- assess patient satisfaction and subjective perceptions of information prescription impact on psychological aspects.

## MATERIALS AND METHODS

The present study was a prospective pilot study, that was conducted for patients with thyroid cancer at IRCCS Regina Elena National Cancer Institute of Rome, a Scientific Institute for Research, Hospitalization and Healthcare and one of the largest public Comprehensive Cancer Centres in Italy. The study was carried out in collaboration with the Oncological Endocrinology Unit and the Digital Library “R. Maceratini.” People eligible for this study were patients aged over 18 years who were referred to our Oncological Endocrinology Unit and had received a new diagnosis of thyroid carcinoma within the past two months.

The study was conducted under the approval of the Local Ethics Committee of IRCCS Regina Elena National Cancer Institute (reference number: RS1035/17) and all patients gave their written informed consent to participate. Patients were consecutively enrolled between January 2020 and December 2021.

After receiving the signed informed consent, Endocrinology Unit clinicians provided health information through “prescriptive information certificates.” Patients with this prescription form were then directed to first access the library, where medical librarians provided scientific and patient education material on thyroid cancer.

The material, which was previously validated by Endocrinology Unit clinicians, included brochures produced by scientific associations and societies, as well as relevant and current evidenced-based health information available from suggested websites, and scientific publications in Italian or English, including a clinical overview of thyroid cancer, its treatment options and its prognosis. Furthermore, patients had the opportunity to request additional information resources on topics of interest, either at enrollment or at a later time by directly contacting the library.

All librarians working at our Institute were trained on the type of materials to provide patients, according to protocol. Periodic meetings between librarians and clinicians took place to collect feedback and ensure consistent patient experience.

After accessing the library and reading the informative material, patients were asked to fill out a pilot-tested questionnaire on overall project satisfaction, subjective evaluation of the project’s usefulness, quality of scientific material received, and self-reported emotions and psychological impact of information prescription, as well as possible impact of the information prescription project on self-management of disease. The questionnaire was created for this study and included both multiple choice and free-text questions (Appendix A, Supplementary Figure 1).

For each patient enrolled in the study, demographic and clinical data were collected from medical records and entered into a database for subsequent analysis. We also collected data on time from study enrollment to first accessing the digital library, topics of additional information requested by patients, and patients’ evaluations captured from questionnaires.

After completing the study, participants were divided into two groups: Group A included patients who accessed the library to receive materials and read and completed the questionnaire; Group B comprised of patients who did not go to the library but received standard information only from their endocrinologist. At least 18 months after enrollment, data on follow-up adherence were collected. Adherence was determined based on the number of missed outpatient visits or cancellations.

Categorical variables of interest were expressed as frequencies and percentage values while continuous variables were expressed as mean ± standard deviation or median and minimum-maximum range, as appropriate. We used the Pearson’s chi-square to assess categorical variables; for continuous variables the Shapiro-Wilk test was used, and the Mann-Whitney test was carried out for non-normally distributed variables via the Statistical Packages for Social Sciences (SPSS version 21.0). p values of <0.05 were considered statistically significant. Since this is a pilot study, no calculation for sample size was performed.

## RESULTS

### Patient Characteristics

In the recruitment timeframe, a total of 101 consecutive patients met inclusion criteria and were enrolled in the study. 82 patients were female (81%) and 19 were male (19%), with a mean age of 49.39 ± 13.59 years. Most patients were affected by papillary thyroid cancer. Most patients (98%) were staged according to TNM 8 edition as stage I. None of the patients had distant metastases at diagnosis.

After surgery (thyroidectomy or emi-thyroidectomy) only clinical and radiological follow-ups were suggested to 87 patients (86%), while for 14 patients (14%) radioiodine therapy (I-131 Therapy) was indicated, in accordance with current guidelines [39, 40].

Patient characteristics are summarized in Table 1.

**Table 1:** Patient characteristics.

	Total patients enrolled (n=101)	Group A (n=26)	Group B (n=75)	*p*
**Age**	49.39 ± 13.59	47.38 ± 13.65	50.08 ± 13.6	0.386*
**Sex**	19 M (19%) 82 F (81%)	3 M (11.5%) 23 F (88.5%)	16 M (21.3%) 59 F (78.7%)	0.386§
**Stage**	Stage I: 99 (98%) Stage II: 2 (2%)	Stage I: 26/26 (100%)	Stage I: 73/75 (97%) Stage II: 2/75 (3%)	-
**Histology**	Papillary TC: 94 (93%) Aggressive tumors: -Aggressive Papillary TC: 3 (3%) -Follicular TC: 3 (3%) -Medullary TC: 1 (1%)	24 (92%) 2 (8%)	70 (93%) 5 (7%)	0.859§
**T**	1A:58 (57%) 1B: 34 (34%) 2:7 (7%) 3: 1 (1%) 4: 1 (1%)	1A: 15 (57.7%) 1B: 10 (38.5%) 2: 1 (3.8%)	1A: 43 (57.3%) 1B: 24 (32%) 2: 6 (8%) 3: 1 (1.3%) 4: 1 (1.3%)	0.842§
**N**	0: 86 (85%) 1A: 6 (6%) 1B: 9 (9%)	0: 22 (84.6%) 1A: 2 (7.7%) 1B: 2 (7.7%)	0: 64 (85.3%) 1A: 4 (5.3%) 1B: 7 (9.3%)	0.887§
**Multi-focality of primary tumour**	No: 80 (79.2%) Yes: 21 (20.8%)	No: 22 (84.6%) Yes: 4 (15.4%)	No: 58 (77.33%) Yes: 17 (23%)	0.578§
**Therapy**	Surgery: 87 (86%) Surgery + I-131:14 (14 %)	Surgery: 22 (85%) Surgery + I-131: 4 (15%)	Surgery: 65 (87%) Surgery + I-131: 10 (13%)	0.752§
**Follow up at our center**	Yes: 91 No: 10	Yes: 20 No: 6	Yes: 71 No: 4	n. a.
**All visits**	76/91 (83.5%)	17/20 (85%)	59/71 (83.1%)	0.633§
**Missed 1 app**	12/91 (13.2%)	3/20 (15%)	9/71 (12.7%)	
**Missed 2 app**	3/91 (3.3%)	0/20 (0%)	3/71 (4.2%)	

Abbreviations: n: number; M: males; F: females; TC: Thyroid cancer; T: primary tumor size (T1A, T1B, T2, T3, T4 according to TNM classification 8 edition); N: lymph node involvement (N0, N1A or N1B according to TNM classification); I-131: radioiodine therapy; – test not performed. App: appointments

* T-test;

§ Chi-squared test.

Tumor stage was expressed in accordance to TNM 8 edition n/a: not applicable

### Health Information-Seeking and Efficacy of Information Project

To assess the need for patient health information, we calculated the percentage of patients who completed all the steps of the health information prescription program.

Among the 101 patients enrolled, 26 patients (26%) visited the library and completed the questionnaire, suggesting a personal need for health information.

Regarding the time from enrolment to first library access, most patients first accessed the library on the day they enrolled (20/26 patients), while 3 patients accessed the library within a week and 3 patients within a month.

Considering gender differences, 23 women (28%) and 3 men (16%) accessed the library. People who accessed the library were younger (mean age of group A: 47.38 years; group B: 50.08 years), although this is not statistically significant.

In comparing the clinical data of people who accessed the library to those who did not, there were no differences between the two groups in disease characteristics with regards to histology, staging and prescribed treatment.

To assess follow-up adherence, we narrowed the analysis to patients with a minimum of 18 months of follow-up at our Oncological Endocrinology Unit (91/101 patients). Other patients were considered lost at follow-up.

Most patients (76/91, 83.5%) attended all scheduled outpatient visits, while a minority (15/91, 16.5%) missed 1 or more appointments. There were no statistically significant differences between the two groups: in group A, 85% of patients (17/20) attended all visits compared to 83.1% (59/71) of group B.

### Patient satisfaction and subjective considerations

Regarding the type of information additionally requested by 18 of the 26 patients, the topics of greatest interest were: nutrition (8, 44%); complementary therapy or rehabilitation (5, 28%); prevention of disease and preventive health measures (2, 11%), rights and legal aspects of the oncological patients (1, 6%); psychological aspects (2, 11%), as reported in Supplementary Figure 2. From the results of the anonymous questionnaire regarding overall satisfaction, 65% (17/26) were very satisfied or satisfied, 23% (6/26) neutral and 12 % (3/26) dissatisfied with their involvement in the project. The information was able to respond effectively to the requests or needs according to most patients (65% of patients, 17/26). Moreover, 69% of patients (18/26) considered health information of good quality (completeness and clarity), 19% (5/26) deemed it sufficiently clear and complete while 12% (3/26) instead regarded it slightly clear and complete. 54% of patients (14/26) claimed the health information prescription was very useful, for 42% (11/26) it was quite useful and for 4% (1/26) a little useful.

In the free text for added patients’ notes, it was observed that most scientific materials were clear and simple, except for the documents written in English.

For most patients (69%, 18/26), the prescription of health information increased awareness and knowledge of their condition and enhanced patient-physician communication, allowing them to ask more detailed questions. For a minority of patients (19%, 5/26), receiving clinical information improved adherence to treatment and enhanced communication with family members about their own disease status, as reported in Appendix A, Supplementary Figure 3.

Regarding the psychological impact, the main emotions felt after receiving information were comfort and encouragement for 69% of patients (18/26), safety and awareness for 88% of patients (23/26), 23% of patients (6/26) replied that information caused a little bit of anxiety and worry, and only 4% of patients (1/26) only a little bit of discouragement (Appendix A, Supplementary Figure 4).

Among personal comments in the free text field, most patients reported satisfaction with the project that enhanced awareness of their own disease and encouraged their health status. Many comments highlighted the project’s utility on diagnostic-therapeutic pathways and disease self-management (Appendix A, Supplementary Figure 5).

## DISCUSSION

Health information prescription belongs in patient-centered health care services; it is an innovative approach that allows to provide appropriate information at the right time to help patients in the management of the disease [41, 42]. Although health information prescription is commonly considered a good practice, objective demonstrations of its effectiveness are scarce.

Previous studies did not report the relationship between various demographic variables and participation in health prescription information. To identify the categories of patients who are most interested in the health information prescription project, we assessed both age and sex. We found that a higher percentage of women accessed the library (28% women versus 16% men) even if not statistically significant. This trend is expected if we consider that generally, women pay greater attention to their health compared to men [43], and have a higher rate of health information-seeking behavior [44]. The lack of statistical significance could be related to a small group of patients.

Socio-cultural factors could influence active participation in information prescription projects. Patients with a lower economic status and socio-cultural level generally seek information less frequently [45]. Unfortunately, data regarding the education level of patients are lacking in our study, albeit our patients reported the English language of scientific texts as a barrier to health information, which confirmed previous data present in literature [3, 46].

Unlike previous studies [47–51], we correlated the information-seeking behavior to the cancer histotype or stage. We found no differences and hypothesize that a tumour diagnosis has the same impact on the information-seeking behavior of patients, regardless of histotype or stage. We also investigated clinical consequences as the impact of prescription information on follow up adherence. The study did not demonstrate any significant changes between participating and non-participating patients in terms of adherence to follow-up care. This may be due the fear of neoplastic recurrence.

Our results, in alignment with previous studies [52], showed patient satisfaction and positive effect perceived by patients on self-care. The questionnaires illustrated that patients perceived an improvement in awareness and knowledge of their disease condition. This resulted in better communication with physicians, in alignment with previous studies[17, 29, 53]. Finally, from a psychological point of view, the main emotions generated from receiving information were comfort, encouragement, and safety, suggesting that, overall information prescription could have a favourable impact on the quality of life of patients affected by endocrine neoplasms.

The main limitations of our study are the small number of enrolled patients who accessed the library, which limits generalizability and may not be sufficient to identify the real effectiveness of the prescription on changes in patient adherence to follow-up programs. There are several possible reasons for low participation, including that the library is physically located outside the Hospital and that many patients were enrolled during the period of the Covid-19 pandemic where many measures greatly restricted access to the library. Another possible explanation is the growing spread of Internet usage in recent decades, [54, 55] including among the elderly, which could reduce interest in seeking other forms of information. As a single-arm study designed to assess patients’ participation in an information prescription program, our study is not randomized.

Future research should consider focusing on other oncological conditions, controlling or adjusting for patients’ education level, and using digital or remote delivery for improving adherence, in addition to having larger sample sizes and investigating other aspects of clinical management.

Nonetheless, the study also has important strengths: this is the first study evaluating the impact of information prescription on adherence to follow-up (instead of only the impact on satisfaction or psychological parameters) and that includes stratification of patients based on demographic and disease-related factors.

Moreover, for the first time, health information prescription study has been carried out in the Italian endocrine oncology setting. This pilot study allowed the research team to investigate the conceptual feasibility of health information prescription in patients affected by thyroid cancer and has contributed to understanding the impact of information prescription services on health-care outcomes. Our study demonstrated an overall satisfaction in thyroid cancer patients, who perceived to have greater understanding of their disease; better communication with physicians; and improvement in self-reported feelings of comfort, encouragement and safety. Based on these promising results, we encourage the application and widespread adoption of this good clinical practice tool in other medical fields, particularly in other cancers.

## INSTITUTIONAL REVIEW BOARD STATEMENT

The study was conducted according to the guidelines of the Declaration of Helsinki, and approved by the Local Ethic Committee of IRCCS Regina Elena National Cancer Institute (reference number: RS1035/17, and date of approval: 12 December 2017).

## Data Availability

All data generated or analyzed are included in this article. Further enquiries can be directed to the corresponding author. Data associated with this article area available at https://gbox.garr.it/.

## SUPPLEMENTAL FILES

**Appendix A:** Survey Questions & Figures
