# Supplementary material for: Impact of health information prescription in thyroid cancer
Source: J Med Libr Assoc. 2026 Apr 13;114(2):116–24. doi: 10.5195/jmla.2026.2191 (PMC13075578; doi:10.5195/jmla.2026.2191)

Appendix A

**Supplementary Figure 1:** Questionnaire

| How satisfied are you with health information project ? | Very satisfied | Satisfied | Neutral | Dissatisfied | Very dissatisfied |
| --- | --- | --- | --- | --- | --- |
|  |  |  |  |  |  |
|  | Very much | Much | Enough | Little | Very little |
| Materials provided were able to respond to your need and requests? |  |  |  |  |  |
| Do you consider text and materials provided of good quality? |  |  |  |  |  |
| Do you consider health information prescription useful? |  |  |  |  |  |
| Which aspects related to your disease and health status do you consider more influenced by information?  Multiple answers allowed | Awareness and knowledge of disease | Patient-physician communication | Communication with family | Adherence to treatment | No benefit |
|  | Other (specify)  …………………………  …………… |  |  |  |  |
| What are the main emotions felt after receiving information?  Multiple answers allowed | Comfort | Discouragement | Safety | Anxiety, worry | Confusion |
|  |  |  |  |  |  |
|  | Other | ………………………………….. | ……………… | …………………………. | ……………….  ………………. |

Write your personal comments in the free box (ideas, suggestions for improvement, critical points, psycho-emotional impact of the project)

| ...............................................................................................................................................................  ...............................................................................................................................................................  ...............................................................................................................................................................  ............................................................................................................................................................... |
| --- |

**Supplementary Figure 2.** Topics of major interest requested by patients.


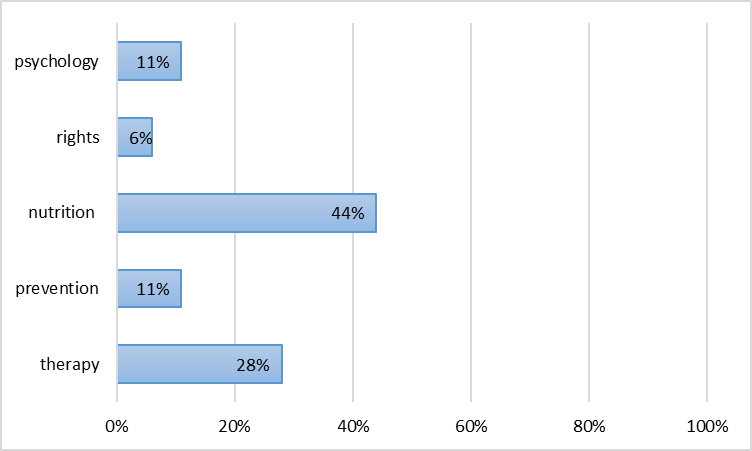


**Supplementary Figure 3.** The aspects of self-management of disease most influenced by health information.

Abbreviations “knowledge” :knowledge and awareness about disease; “comm physician”:communication with physician; “comm family”:communication with family members; “adherence”: adherence to treatment


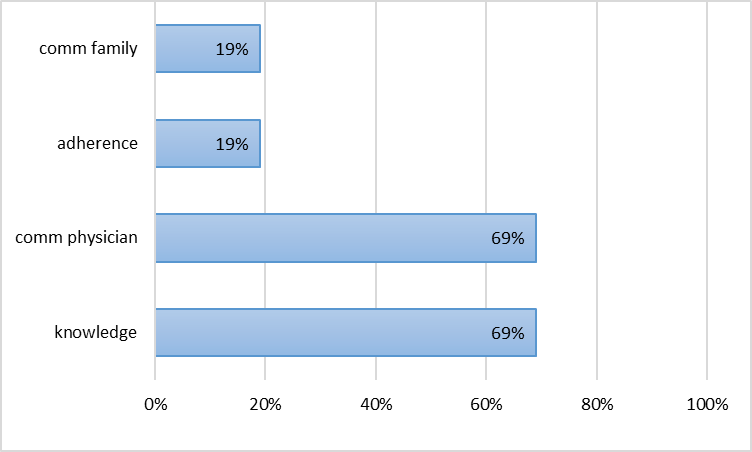


**Supplementary Figure 4**. Psychological impact of prescription information. The main emotions felt are reported in this figure.


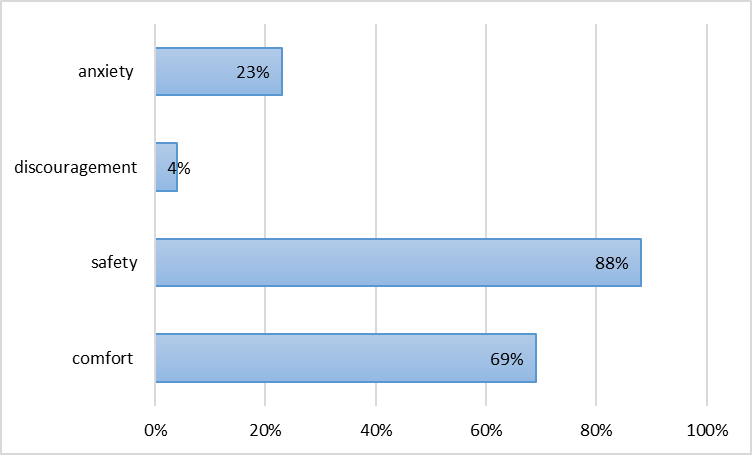


**Supplementary Figure 5** Word cloud: visual representation of keywords used in patient comments. The most frequent keywords are represented in the figure in larger size than the less frequent ones.


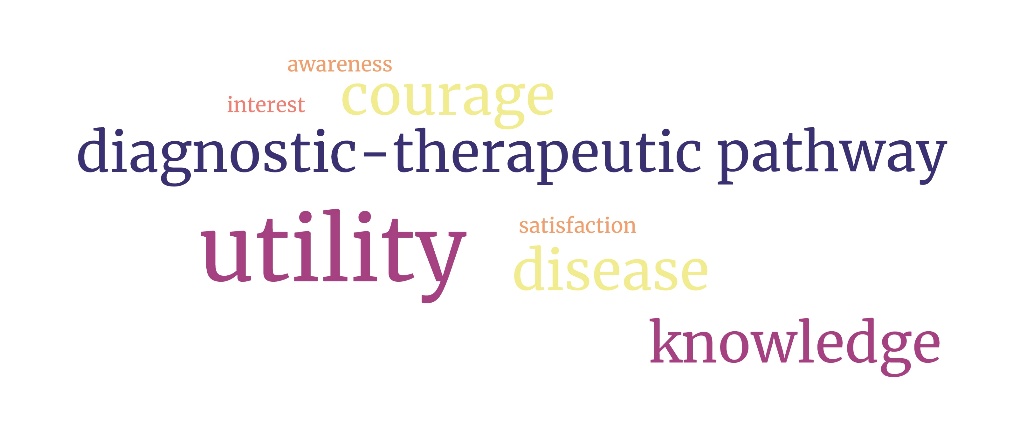

Supplement: Supplementary file 1 — Appendix A: Survey Questions & Figures [file jmla-114-2-116-s01.docx]
